# Reliability and criterion validity of the concept 2 SkiErg™ to assess 1,000-m on-snow, time trial performance—a case study

**DOI:** 10.3389/fspor.2025.1631229

**Published:** 2025-10-17

**Authors:** Brendan J. O'Brien, Ryan Worn, Brad Clark, Matt Spencer

**Affiliations:** ^1^ Federation University Australia, Institute of Health and Wellbeing, Mt Helen, VIC, Australia; ^2^ Research Institute for Sport and Exercise, University of Canberra, Canberra, ACT, Australia; ^3^ Department of Sports Science and Physical Education, Faculty of Health and Sports Sciences, University of Agder, Kristiansand, Norway

**Keywords:** cross-country skiing, double poling, ergometry, physical testing, coefficient of variation (CV)

## Abstract

**Objectives:**

This study investigated the reliability and criterion validity of the Concept 2 SkiErg™ to assess 1,000-m on-snow, time trial performance using the classical double poling technique.

**Methods:**

Ten athletes (5 males and females) from a National cross-country ski team participated in the study and completed a 1,000-m time trial on snow, as well as two 1,000-m time trials on the Concept 2 SkiErg™ in a temperature-controlled room during a 4-day training camp.

**Results:**

There was a significant decrease in time from test 1 to test 2 of 4.87 s [238.3 ± 26.1 vs. 233.4 ± 23.9 s; 95% limits of agreement (LoA): −5.5, 15.3]. The Concept 2 SkiErg™ time-trial had a coefficient of variation (CV) of 1.6% and the standard error of measurement was 3.8 s. When compared to the on-snow time-trial, the Concept 2 SkiErg™ time-trial demonstrated a mean bias of 20.7 s (95% LoA; 11.6, 29.8) and the concordance correlation coefficient was 0.72.

**Conclusion:**

The Concept 2 SkiErg™ demonstrated excellent single-trial reliability. However, there was significant proportional bias in the Concept 2 SkiErg™ relative to the on-snow test and agreement between the two was relatively poor. Research using a larger sample and different trial durations is required to further validate the Concept 2 SkiErg™ for cross-country skiing performance testing.

## Introduction

Cross-country skiing is an Olympic and global sport contested by over 50 nations (1). It is a physically demanding sport, requiring whole body strength and power, high and sustained aerobic energy turnover and repeated work bouts above peak oxygen uptake (V̇O_2peak_), interspersed with short recovery periods (2, 3). However, standardised testing and assessment of cross-country skiing performance can be challenging due to unpredictable snow fall and snow conditions particularly in regions or altitudes most affected by climate change (4). Therefore, indoor testing is important element of monitoring cross country ski performance.

Specificity of an exercise test to actual field performance is an important concept in sport performance assessment (5). Additionally, knowledge of the reliability of a performance test is also critical to discern genuine intervention effect from random error. Studies reporting the reliability and validity of cross-country ski tests are limited. The double poling motion of cross-country skiing is a unique movement that cannot be precisely simulated by treadmills, arm cranking and cycle ergometers. Consequently, specific tests have been developed to assess during the double poling motion. The Concept 2 rowing ergometer™ has been modified to test the double-poling technique and shows excellent reliability to determine V̇O_2peak_ (*r* = 0.99) (6) and capability to determine V̇O_2peak_ in the field (7).

To further improve the specificity of cross-country skiing test the Concept 2 SkiErg™ ergometer has been developed. The Concept 2 SkiErg™ has excellent reliability in V̇O_2peak_ determination (coefficient of variation of 1.7%) (8). The Concept 2 SkiErg™ also has very strong correlations with a treadmill ski-striding protocol for V̇O_2peak_ (*r* = 0.95) (8). However, the test-retest error (reliability) for 1,000-m time trial performance and capability of the Concept 2 SkiErg™ to discern genuine intervention effect from random error and the criterion validity to assess on-field snow performance has not been reported. Therefore, this studies objective is to determine Concept 2 SkiErg™ reliability and criterion validity against on-snow performance.

## Method

### Participants

Ten members (5 males and 5 females) of a national cross-country ski team participated in this study. The caliber of athletes were Tier 4: Elite/International and Tier 3: Highly Trained/National Level. The overall average age, height and body mass of the participants was 20.4 ± 3.8 years, 179.8 ± 9.7 cm and 70.0 ± 10.5 kg. The study was approved by the University Ethics committee (A12-059). Informed written consent was provided by each participant during an on-snow training camp held over 4 days at an elevation of ∼1,200 m.

### Protocol

**Figure 1:**
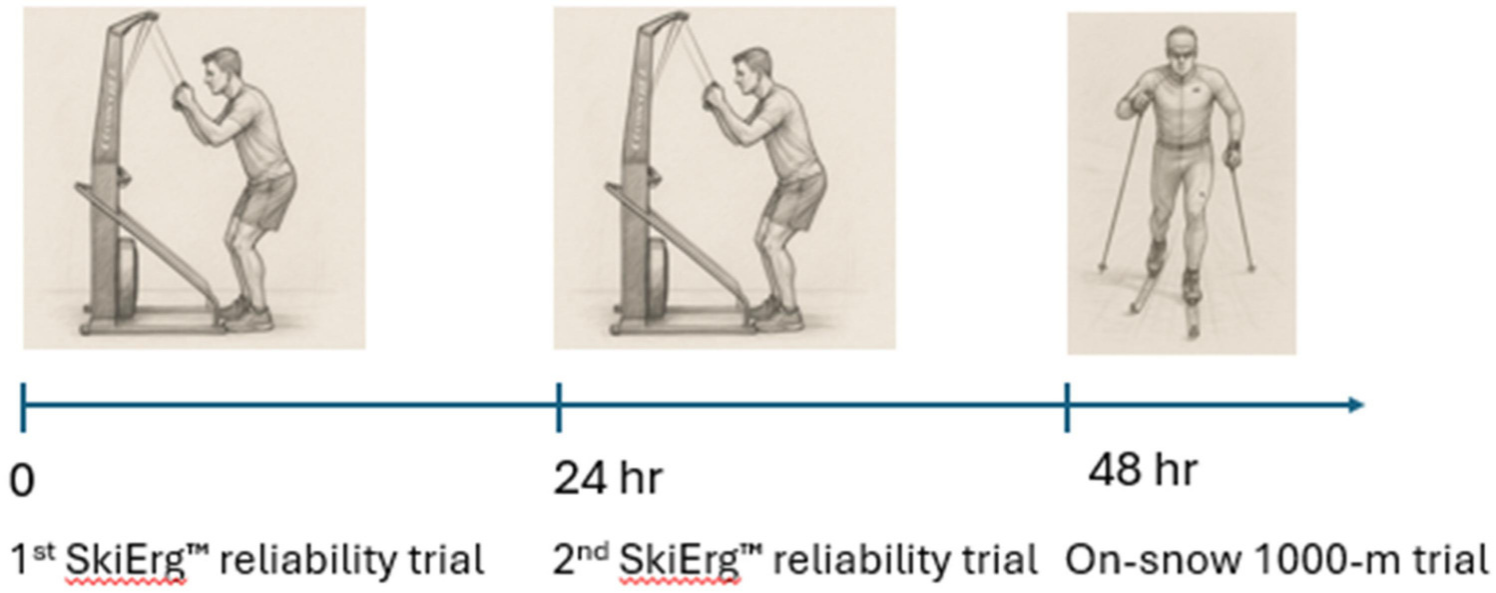
The chronological sequence of experimental design.

Athletes completed two Concept 2 SkiErg™ tests in a temperature-controlled room (18°C) and at an equivalent altitude to the on-snow test (∼1,200 m) over the 4 day-camp in the mornings on consecutive days for 24 h recovery between tests. All participants had prior experience using the Concept 2 SkiErg™ in training. All participants completed a self-selected intensity 10-minute warm-up before the 1,000-m time trial test on the Concept 2 SkiErg™ ergometer. The damper was set at 5. The time trial test was performed 5 min after the warm-up with participants instructed to race as fast as possible. On the following day the athletes completed a 1,000-m maximal flat terrain effort time trial on-snow over flat terrain (±5 m altitude change) using the classical double poling technique. The 1,000-m time trial was performed on a straight-line point-to point course facing west. Athletes used their own ski equipment. Waxing products were supplied by the team's service personnel. The waxing protocol was performed by two service personnel and involved cleaning the ski base with a specialized wax remover, applying a hydrocarbon base wax to saturate and protect the ski base, and applying in a high-fluorocarbon (HF) glide wax. After cooling, wax was scraped and brushed to refine the glide surface. A 1,000-m trial was chosen for its similarity to a competitive sprint cross-country ski race (1). Athletes started each time trial individually on 60 s intervals, in sequence of anticipated time trial performance (slowest to fastest). The air temperature was stable (1.2 ± 1°C) at the time. The barometric pressure at the time was 1,022 mbar, with no precipitation and high visibility with an estimated westerly breeze of approximately 7 km/h. Athletes wore an accelerometer and GPS unit (MinimaxX™, Team Sport Model, Catapult, Australia) to record completion time and poling cadence (9). The natural snow depth on the day of the on-snow time trial was estimated to be 120 cm. Figure 1 shows the timeline of the project.

### Statistics

All analyses were conducted in R statistical software (v4.2.1; R Core Team 2022) (10). The reliability of completion time from the Concept 2 SkiErg™ test was determined from the two indoor trials and determined using the mean difference, coefficient of variation (CV), standard error of the measurement, intraclass correlation coefficient, and 95% limit of agreements (LoA).

The validity of completion time from the second Concept 2 SkiErg™ test was evaluated against completion time and poling cadence from the on-snow test. Validity of the Concept 2 SkiErg™ test was determined by calculating the mean difference, 95% LoA, and concordance correlation with the on-snow test. Additionally, proportional bias was assessed using linear regression of the differences against the mean of the two tests. Analyses were conducted using the “SimplyAgree” package (11).

Intraclass correlation coefficient reliability was interpreted as poor (ICC <0.5), moderate (0.5 ≤ ICC < 0.75), good (0.75 ≤ ICC < 0.90) and excellent (ICC ≥ 0.90). The concordance correlation coefficient was interpreted as: poor (<0.90), moderate (0.90–0.95), substantial (0.95–0.99) and almost perfect (>0.99) (12). Reliability and validity data were visualised as correlation and Bland–Altman plots using the “ggplot2” package (13).

## Results

**Figure 2:**
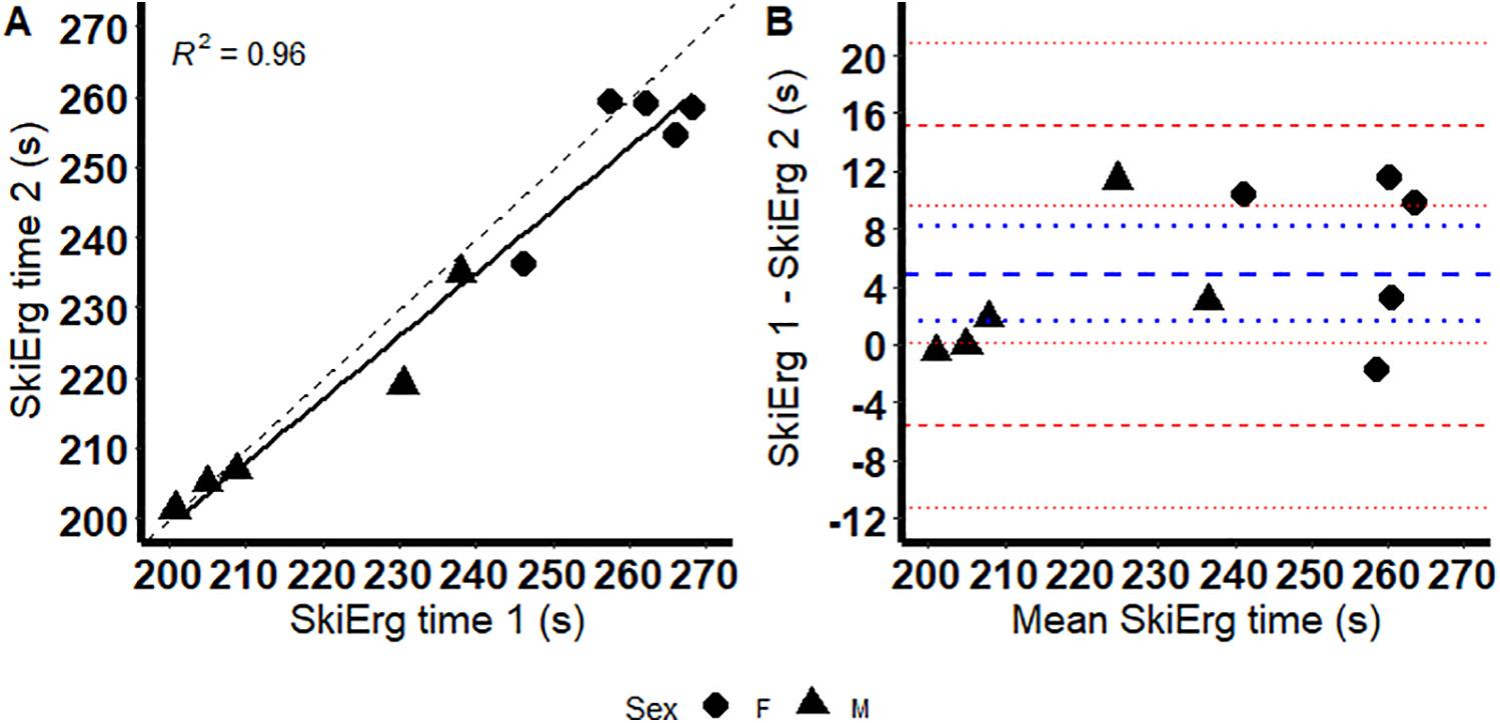
Correlation (sold line) plot for completion time between the first and second test from the concept 2 SkiErg™ **(A)**; dashed line represents the line of identity. Bland–Altman plot showing the mean difference (thick blue dashed line), 95% confidence interval (95% CI) for the mean difference (dotted blue line) and the 95% limits of agreement (thick dashed red lines) and their 95% CIs (dotted red lines) of the first and second test from the Concept 2 SkiErg™ **(B)**.

Completion time reliability of the 1,000-m Concept 2 SkiErg™ is presented in Table 1. The second test was approximately five seconds faster than the first test (Figure 2), which was statistically significant. The ICC of 0.98 revealed excellent reliability. Poling cadence was not significantly different between the first and second tests (52.4 ± 7.3 vs. 52.9 ± 4.8 poles/min; *p* = 0.7447).

**Table 1:** Reliability of the concept 2 SkiErg™ time trial in determining time to completion.

Variable	Time to completion (s)	
	Test 1	Test 2
Mean ± SD	238.3 ± 26.1	233.4 ± 23.9*
Range	200.7–268.3	201.2–259.3
Mean difference [95% CI]	4.87 [1.08, 8.66]	
95% LoA	−5.5, 15.3	
SEM	3.8	
CV (%)	1.6%	
ICC (r)	0.98	

CV, coefficient of variation; ICC, interclass correlation coefficient; LoA, limits of agreement; SD, standard deviation; SEM, standard error of measurement.

* Denotes test 2 significantly faster than Test 1 *p* = 0.0174.

**Figure 3:**
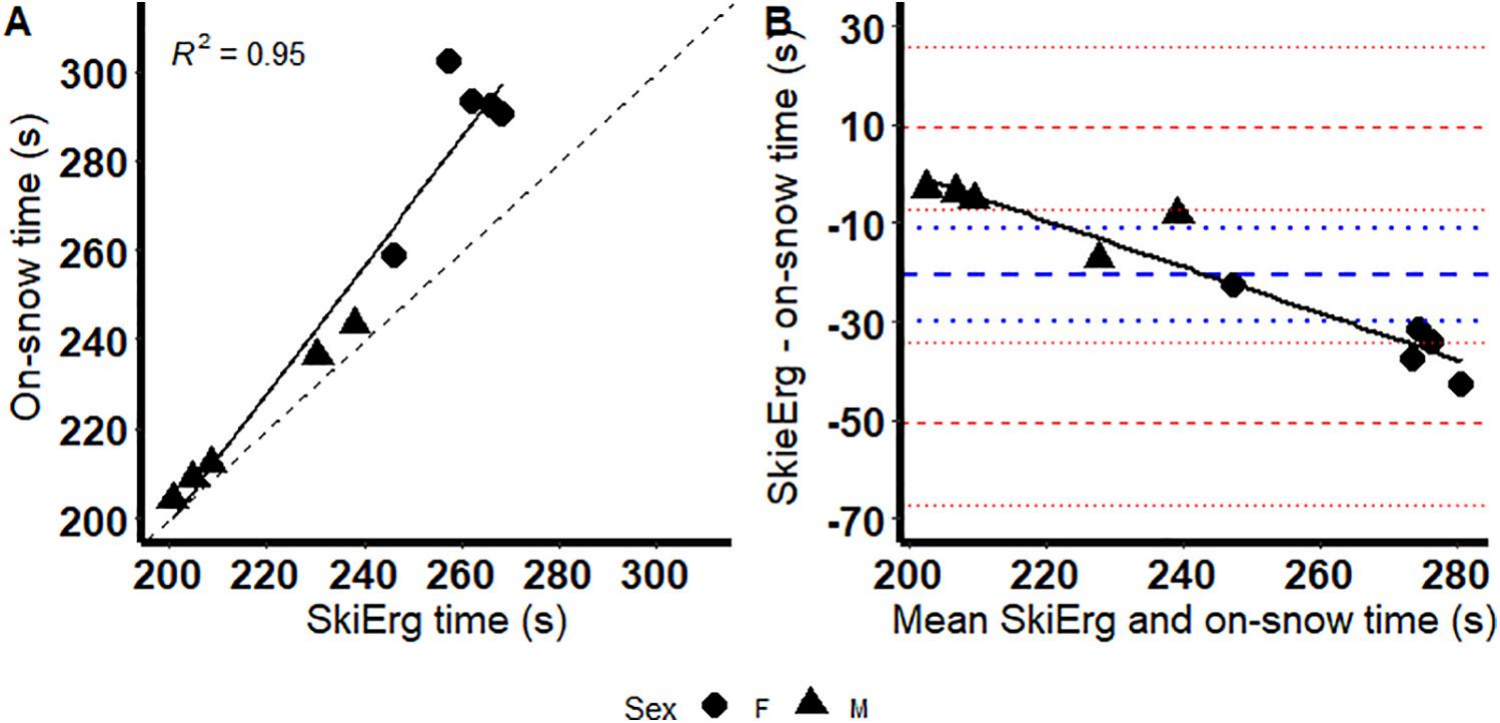
Correlation (solid line) plots between the SkiErg™ and the on-snow test for completion time **(A)**; dashed line represents the line of identity. Bland–Altman plot showing the mean difference (thick blue dashed line), 95% CI for the mean difference (dotted blue line) and the 95% limits of agreement (thick dashed red lines) and their 95% CIs (dotted red lines) between the SkiErg™ and the on-snow test **(B)**; solid black line represents linear regression of the differences on the mean.

The criterion validity of the 1-km Concept 2 SkiErg™ test to assess 1-km on-snow time to completion is presented in Table 2. The concordance correlation coefficient between the tests of 0.72 is rated as poor. The data indicates the average Concept 2 SkiErg™ time trial was significantly faster than the on-snow test. There was significant proportional bias between the Concept 2 SkiErg™ test and 1,000-m on-snow time (Figure 3), indicating that the bias between the methods increased with the magnitude of measurement. However, the poling cadence was not significantly different between the Concept 2 SkiErg™ and on-snow test (54.9 ± 2.2 vs. 52.6 ± 6.0 poles/min; *p* = 0.304).

**Table 2:** Comparison of the SkiErg™ with an on-snow test for measuring 1,000-m completion time.

Variable	Time to completion (s)	
	SkiErg™ test 2	On-snow
Mean ± SD	233.4 ± 23.9	254.1 ± 38.6*
Mean difference	−20.7	
95% LoA	−11.6–29.8	
CCC (r)	0.72	

CCC, concordance correlation coefficient; LoA, limits of agreement.

* Denotes SkiErg™ significantly faster than On-snow test (*p* = 0.0037).

## Discussion

The purpose of the current study was to determine the reliability of the Concept 2 SkiErg™ and criterion validity relative to a 1,000-m on-snow double poling time trial. The standard error of measurement of 3.8 s (CV of 1.6%) indicates the Concept 2 SkiErg™ possesses excellent reliability. Practically, a reliable test should consistently identify genuine and meaningful changes in performance and detect the smallest worthwhile change. The smallest worthwhile change represents the minimum change in performance that is practically meaningful for performance. The smallest worthwhile change is calculated from the standard deviations multiplied by a small effect size (0.2). We calculated this as 4.8 s (SD of 23.9 s × 0.2). We determined the Concept 2 SkiErg™ to have a standard error of measure of 3.8 s. Consequently, the Concept 2 SkiErg™ is likely to determine the smallest worthwhile change in cross country ski time trial performance in most circumstances (14, 15). The reliability of the Concept 2 SkiErg™ to determine 1,000-m time trial performance complements the findings that it also has excellent reliability in VO_2 peak_ determination (8). While the data is reliable, there was a significant (*p* < .05) difference between test 1and 2, with test 2 being significantly faster by ∼5 s. This most likely occurred due to a learning effect from the first test to the second test (14–16). A learning effect often occurs in tests following the initial test as athletes learn to improve their pacing and possibly become biomechanically more efficient in their technique and action (14–16). Consequently, it is recommended that athletes practice specific time trial tests at least twice before official results are recorded to minimize the impact of learning affecting the test reliability (14, 15).

The criterion validity of the Concept 2 SkiErg™ was relatively poor with a concordance correlation of 0.72 between Concept 2 SkiErg™ and on-snow performance. Furthermore, Concept 2 SkiErg™ performance was significantly faster than on-snow performance and there was significant proportional bias which suggests the Concept 2 SkiErg™ increasingly underestimated completion time as on-snow completion time increased. Accordingly, Concept 2 SkiErg™ may not be an appropriate tool to predict on-snow cross country skiing performance, particularly for slower skiers.

Our data show that men had superior performance times than women. The time trial difference men and women can be attributed to men's larger body mass of the male skiers corresponding to a 55% higher power output compared to their female counterparts (17). The higher power output of men is consequent to inherent biological differences, including higher testosterone, greater hemoglobin mass and a larger body with more muscle mass. These differences allow for a greater delivery of aerobic and anaerobic energy and, consequently, higher power capability for men than women (17).

### Limitations and future research

This study had a low sample size of elite athletes. A larger sample and more heterogenous sample (cross-country skiing capability) is required to identify possible heteroscedasticity in the data as performance standard improves. The study was limited to 1,000-m performance due to convenience and was not counter-balanced due to the practical implementation of a training camp with national team athletes. Due to time constraints a familiarization of the tests prior to their investigation was not conducted which led to bias in Concept 2 SkiErg™ time trial performance. Cross-country skiing distances vary widely, from short sprints of 1,000-m to events longer than 50 km. Research to determine the Concept 2 SkiErg™ capability to predict time trial performance in longer distances is required. There are technical/biomechanics differences between Concept 2 SkiErg™ and on-snow cross country performance (14). After each pole stroke the ergometer cord recoils rapidly. This recoil pulls the participant's hands back up and aids returning to the start poling position. This is different action to skiing, where the athlete brings back their hands and poles themselves after each pole stroke, against gravity and unaided. Development and investigation of an ergometer that more closely replicates cross-country skiing is required. Additionally, the indoors (18°C) and outdoors temperature varied (1.2 ± 1 °C) and should be controlled to eliminate temperature effects on performance. Future research should control sleep, diet, prior exercise in the days preceding testing and control for caffeine intake on the experimental days. Additionally future research could investigate if repeated short-duration SkiErg™ tests (e.g., weekly 1,000-m time trials) improves individual-level predictive utility over time, despite modest (or poor) criterion validity from a single trial.

It should be noted that the high-fluorocarbon (HF) glide wax used in this study is no longer permitted under current FIS regulations. Consequently, the observed performance outcomes may not fully translate to competitions using only currently legal waxing products, and differences in glide performance could alter the practical application of these findings.

### Practical applications

The Concept 2 SkiErg™ was determined to have excellent reliability and is likely to determine the smallest worthwhile change in 1,000-m cross country ski time trial performance in most circumstances.

### Conclusion

The Concept 2 SkiErg™ is a reliable test of 1,000-m time trial performance. The Concept 2 SkiErg™ has poor concordance correlation with 1,000-m on-snow performance and may not be an appropriate tool to predict on-snow cross country skiing performance, particularly for slower skiers.

## Data Availability

The raw data supporting the conclusions of this article will be made available by the authors, without undue reservation.
